# Acceptability and stakeholders perspectives on feasibility of using trained psychologists and health workers to deliver school-based sexual and reproductive health services to adolescents in urban Accra, Ghana

**DOI:** 10.1186/s12978-018-0564-x

**Published:** 2018-07-05

**Authors:** Philip Teg-Nefaah Tabong, Ernest Tei Maya, Terence Adda-Balinia, Dela Kusi-Appouh, Harriet Birungi, Placide Tabsoba, Philip Baba Adongo

**Affiliations:** 1 Department of Social and Behavioural Sciences, School of Public Health, University of Ghana, Accra, Ghana; 2 Department of Population, Family and Reproductive Health, School of Public Health, University of Ghana, Accra, Ghana; 3 Population Council, Accra, Ghana; 4 Population Council, City, Nairobi, Kenya

**Keywords:** Sexual and reproductive health, Adolescents, Health workers, Psychologists, School-based adolescents health services, Feasibility, Acceptability, Ghana

## Abstract

**Background:**

Adolescent sexual and reproductive health is recognized as a key developmental issue of international concern. However, adolescents’ access to sexual and reproductive health (SRH) information and services is largely inadequate in sub-Saharan Africa. With increasing enrollment in schools, this could be an avenue to reach adolescents with SRH information and services. This study was therefore conducted to assess the acceptability and stakeholders’ perspectives on the feasibility of using health workers and trained psychologists to provide school-based SRH services in Ghana.

**Methods:**

Fourteen (14) focus group discussions (*N* = 136) were conducted; 8 among adolescents aged 12–17 years (4 boys, 4 girls groups), 4 among parents (2 males, 2 females groups) and two among mixed teacher groups. We also conducted 18 in-depth interviews with teachers, managers of schools, health workers, clinical psychologists, as well as adolescent SRH program managers in the Ghana Health Service, Ghana Education Service, UNICEF, UNESCO and National Population Council, Ghana. We audio-recorded all interviews and took field notes. Interviews were transcribed and transcripts imported into NVivo 11 for analysis using grounded theory approach to qualitative data analysis.

**Results:**

Many respondents reported that it was challenging for parents and/or teachers to provide adolescents with SRH information. For this reason, they agreed that it was a good idea to have health workers and psychologists provide SRH information and services to adolescents in school. Although, there was general agreement about providing SRH services in school, many of the respondents disagreed with distribution of condoms in schools as they believed that availing condoms would encourage adolescents to experiment with sex. While majority of respondents thought it was acceptable to use psychologists and health workers to provide school-based sexual and reproductive health to adolescents, some teachers and education managers thought the implementation of such a program would oppose practical challenges. Key among the challenges were how to pay for the services that health workers and trained psychologists will render, and the availability of psychologists to cater for all schools.

**Conclusion:**

Stakeholders believe it is feasible and acceptable to use trained psychologists and health workers to deliver school-based SRH information and services in the Ghanaian school context. However, provisions must be made to cater for financial and other logistical considerations in the implementation of school-based SRH programs.

## Plain English summary

Adolescents face challenges accessing reproductive and sexual health services. The lack of access to these services make some adolescents engage in unhealthy sexual and reproductive health behavior. Globally, there has been a drive towards finding innovative ways to increase access to sexual and reproductive health services. With increasing enrollment of adolescents in school, it presents an opportunity to explore ways to use the school environment to increase access to sexual and reproductive health services. Many of the behaviors that adolescents put up are caused by psychological factors. This therefore calls for some level of expertise in psychology and health to be able to provide the needed assistance. We therefore conducted a study among teachers, students, managers of educational institutions, health care workers engaged in adolescent’s sexual and reproductive health services and development partners on their views about using trained health workers and psychologists to deliver the service.

Our study found that students, teachers and various stakeholders believe the current strategies used to provide adolescents with sexual and reproductive health services were inadequate and therefore felt that it was better to use trained psychologists and health workers to deliver these services to adolescents in school. However, there was the need to do further consultation and put in measures to cater for logistical issues and incentives for the people to provide these services.

In conclusion, stakeholders in this study believe it was feasible and acceptable to use psychologists and health workers to deliver school-based sexual and reproductive health services.

## Background

Adolescent sexual and reproductive health (ASRH) is recognized as a key developmental issue of global concern. While reproductive health information and service delivery have been identified as necessary programs for decades, availability of such programs for adolescents has only recently been more endorsed. The International Conference on Population and Development (ICPD) held in 1994 and the Fourth International Conference on Women held in 1995 endorsed the rights of young people to sexual and reproductive health (SRH) information and services [1]. Health and development professionals and policy makers have built on this consensus to formulate and deliver needed programs that are specially designed to meet adolescents’ developmental needs. Over the past decades, the framework of adolescent friendly health care has been used to better orient health services to the needs of young people. Initially described by the World Health Organization (WHO) and largely focused on primary health care in low-income countries, there is growing appreciation of the framework’s potential to promote quality health care to adolescents in high-income countries within specialized health services [2].

Despite these commitments, SRH information and services remain largely inadequate in sub-Saharan Africa. Many adolescents face an early sexual debut and many face difficulties in obtaining SRH services [3–7]. Adolescents are also typically poorly informed about how to protect themselves from pregnancies and sexually transmitted infections (STIs), which threaten their health and survival [5, 7]. A nationwide survey in Ghana found that 14% of females aged 15–19 years had begun child bearing. Of these 14%; about 11% have had a life birth and 3% were pregnant at the time of the survey [8]. Contraceptive use are also low as another nationwide survey of 1037 adolescents shown an overall prevalence of 18.3% comprising 14.6% of modern methods and 3.7% of traditional methods [9]. Similarly, another study reported that unsafe sexual and unsafe abortion practices was common and viewed as normal [10].

Comprehensive Sexuality Education (CSE), which is defined as age-appropriate, culturally relevant, scientifically accurate, realistic, and nonjudgmental education about sexuality and relationships, offers an appropriate platform to provide adolescents with critical SRH information [11]. CSE has been reported to have significant impact in addressing the SRH needs of adolescents in other countries [12–14]. Reproductive health services incorporate a range of activities (e.g. health promotion, prevention, early diagnosis, treatment and care, rehabilitation). In Ghana, Population and Family Life Education (POP/FLE) a form of CSE was introduced into the basic education system of the Ghana Education Service (GES) between 1973 and 1979 on a pilot basis, re-activated in 1987 and again in 1994. Due to the lack of a comprehensive in-school approach to this the POP/FLE has been unable to achieve its goal as many adolescents have low knowledge on SRH [15–17].

Guidance and counselling services were established in 1976. The Ghana government came out with a policy, through a directive issued by the Ghana Education Service (GES), for the establishment of guidance and counselling in the nation’s second cycle institutions [18]. The School Health Education Programme (SHEP) was also established in 1992 as a follow-up action on Ghana’s commitment to the Jomtien World Declaration on Education for all and her ratification of the United Nations Convention of the Rights for the Child. It was established as a joint mandate to the Ministry of Education (MoE) and the Ministry of Health (MoH). The SHEP program was to facilitate the provision of health education to the door steps of school children, foster early detection of disability and to inculcate into them health promotion habits, attitudes and values. Despite all these interventions which are mostly handled by trained teachers, evidence from a study showed the desire of adolescents towards receiving accurate and more comprehensive reproductive and sexual health information and service in schools. An initial study conducted by Population Council under the Strengthening Evidence for Preventing Unintended Pregnancies (STEP UP) project in 2012 showed that 80% (both sexes) of the adolescents interviewed indicated the need for more information on reproductive health [19].

In developing countries parents, school teachers, mass media, social media and peers have been reported as the main sources of information on sexual and reproductive health [15, 20–22]. However, these channels have been reported to have challenges in meeting the informational needs and services of adolescents [15, 22, 23].

In Ghana, adolescents constitute about a quarter of the total population and their population is expected to continue to increase [24]. Despite this, many have been reported to have low and inaccurate knowledge about their sexual and reproductive health (SRH), [25–27]. A study among adolescents in Central Region of Ghana by Owusu, Blankson & Abane [20] showed that the major sources of information on SRH to adolescents were their friends (30%), radio/television (26%) and parents (13%) [20]. However, information adolescents receive from these sources have been reported in many instances to be inaccurate, not focused on adolescent needs and also limited in scope [15]. This lack of knowledge has led to high incidence of sexually transmitted infections (STIs) and unplanned pregnancy among adolescents in Ghana [28]. Beyond the lack of knowledge, access to reproductive health services such as contraceptives, treatment for STIs and safe abortion services have been a challenge in Ghana [8]. Socio-cultural barriers and lack of adolescent friendly SRH services leads to poor patronage of such services even when they are available [3, 29, 30].

Studies have also showed that the behavior during the period of adolescents are triggered by psychological factors [31, 32]. Many health compromising behaviors, such as unsafe sexual practices, use of tobacco, alcohol and other psychoactive substances, that begin during adolescence have profound consequences for their health and development and also long term wellbeing [2, 33, 34]. The use of nurses to provide ASRH services in schools and psychologists to provide counselling and psychological support to adolescents is believed to be essential in addressing the problem adolescent face. Nurses will also diagnose and treat STIs among adolescents and liaise with formal health facilities to provide comprehensive abortion services to reduce the negative effects of unsafe abortions as has been reported in a study [35–38]. Nonetheless, the use psychologists to provide school-based health services has not been reported in literature. Currently in Ghana psychologists are mainly employed in academia, industry, health facilities especially mental health facilities and the private sector. Although data is not readily available on the regional distribution of psychologists, records available at the Ghana Psychologist Council show there are about 116 clinical, 35 counselling and 10 educational psychologists [39]. Nonetheless, there are several trained psychologists who are not registered with the council and not practicing despite the fact that the Health Professionals Act, 2013, (Act 587 of 2013), Domestic Violence Act, 2007 and Mental Health Act 2012 requires members to be registered before they can be employed in the formal sector [40].

From our review of literature, no study has been conducted to assess the stakeholders’ beliefs about the feasibility and acceptability of using trained psychologists and health workers to provide these services. The increase in enrollment in school in recent times makes the school environment presents an opportunity to reach students with CSE and SRH services. Thus, we conducted this study to assess the acceptability and stakeholders’ perspectives about the feasibility of using health workers and trained psychologists to provide SRH services in schools in Ghana.

## Methods

### Study design

We conducted a qualitative study comprising of in-depth interviews and focus group discussions (FGDs) with junior high school students, teachers, school managers and other stakeholders to assess their view on the feasibility of using psychologists and health professionals to deliver SRH information and services to in-school adolescents. We adopted grounded theory approach to qualitative research in this study. In grounded theory approach to qualitative research, the researcher undertakes a research and develops a theory or theoretical framework that is grounded on the data [41, 42]. The approach was used to identify the contextual issues and approaches to be adopted in Ghana.

### Study area

The study was conducted in Nima in the Ayawaso sub-metropolis of the Accra Metropolitan Assembly. The Accra Metropolitan Assembly (AMA) has a total population of 1,665,086 representing 42% of the region’s total population. Females constitute about 51.9% of the population [43]. The Metropolis, which is entirely urban (100%), has a fairly youthful population as about 42.6% of the population are children under 15 years [24]. Nima, the study site reflects the metropolitan structure as it serves as a hub for migrants. Forty-seven percent of residents are migrants; this proportion is the highest in the Greater Accra region [44]. Early marriage is common in the area [44]. There were 14 Junior High Schools in Nima with a total student population of 2492 aged between 12 and 17 years [45].

### Recruiting of respondents

We recruited five categories of respondents into this study; students, parents, teachers, educational managers and representatives of development partners. The district education office supported the recruitment of students. We selected two out of the 14 junior high schools based on two main criteria: recorded high school dropout rates due to teenage pregnancy and presence of trained ASRH teachers. From the two selected schools, 79 adolescent students (39 females and 40 males) aged 12–17 years were recruited to participate in the study. We obtained the list of students who have not been absent from school over the term and students who have attended courses which included some SRH education. From this list, we selected the actual participants based on their willingness to participate. These students participated in age and sex-specific FGDs: female students aged 12–14 years; female students aged 15–17 years; male students aged 12–14 years; and male students aged 15–17 years.

We recruited parents of students in the two schools who regularly participated in school activities such as Parent Teacher Association (PTA) meetings. This was done to ensure that we selected parents who were engaged in school-related activities. Teachers and head teachers assisted the research team to purposively select parents who met this inclusion criterion. Purposive sampling was also employed to select key informants in education and health who were in-charge of adolescent SRH issues at various levels. We contacted Ghana Education Service for contacts numbers of the managers who oversee adolescent reproductive health services in the Metropolis. We then reached out to them and booked an appointment for the interview. For representatives of development partner, the research team visited the various offices and the focal persons were informed about the study and a consent obtained before they were interviewed.

## Data collection strategy

### In-depth interviews

A semi-structured in-depth interview (IDI) guides was developed or the data collection. The same IDI guide was used in interviewing education managers, health workers, psychologists and development partners. The interview guide for these stakeholders focused on participants’ views about school-based adolescent SRH services, how such services should be delivered, whether using trained psychologists and health workers to deliver these services was feasible, and the ranges of services that should be provided. Interviews were conducted in private and lasted between 45 and 60 min. All interviews were audio-recorded.

In all 18 key informants were selected and interviewed. In the educational sector, we recruited people such as basic education coordinators, school health coordinators, heads of basic schools, and teachers. We also interviewed key personnel in-charge of adolescents’ SRH from the Ghana Health Service at sub-metropolis, metropolis and national levels. In addition we interviewed representatives from development partners whose activities include adolescent sexual and reproductive. Table 1 summarizes the number of different key informants who participated in the study.

**Table 1:** Summary of Key Informants

Category of Respondent	Number interviewed
Ghana Education Service Program Managers	5
Heads of Basic Educational Schools	3
Ghana Health Service ASRH Program Managers	4
Population Council Representative	1
Members of Ghana Psychologist Association	2
UNESCO Representative	1
Teachers	2
Total	18

### Focus group discussions

Semi-structured FGDs were conducted to explore normative views about SRH information received while exploring the feasibility and acceptability of the proposed solution. Different FGD guides were designed for students, teachers and parents. The topic guides explored adolescents’ and parents’ normative views on the following key broad areas: SRH information and services delivery preferences; viability and acceptability of providing in-school reproductive health services by health workers; delivering sexual and reproductive health care using psychologists and health workers at the school level. However, during the FGDs, inductive probing was conducted on emerging new areas. Two research assistants conducted the FGDs, one moderated the discussions while the second took detailed notes. The note taker took notes on the group dynamics and body language of participants as well as seating arrangements to supplement and guide the interpretation on the transcripts. All discussions were audio-recorded. Fourteen (14) separate group discussions were held: eight among adolescents (four per school), four among parents (males and females separately in each school) and two among teachers (one per school). Each group comprised 9 to10 participants and the discussions lasted for approximately one hour and thirty minutes. Table 2 provides the summary of the participants in the FGDs.

**Table 2:** Summary of Participants in FGDs

Participants	Number of FGD	Number of Participants
Female Students		
12–14 years	2	19
15–17 years	2	20
Male Students		
12–14 years	2	20
15–17 years	2	20
Parents		
Males	2	20
Females	2	19
Teachers		
Mixed male/female groups	2	18
Total	14	136

### Data analysis

The audio-recordings and field notes were transcribed verbatim in English using Microsoft Word. The researchers read through transcripts and noted the emerging issues from the data. These were transformed into a codebook. The codebook contained various themes in the data, their definition and where such themes should be used in coding the data. The codebook was reviewed by the research team, imported into QSR NVivo 11 as nodes. The transcripts were also imported into QSR NVivo 11 for analysis. We first classified the data sources (FGDs, and IDIs) and assigned the classifications to the data sources. Various respondents were captured as cases and attributes such as designation, gender and age assigned to the respondents (cases). This allowed us to run queries in NVivo to quantify the qualitative data sources and cases. Grounded theory was adopted for the analysis of the data. This approach involved three interrelated steps; open coding, axial coding and selective coding [46]. For the start, line-by-line coding of responses from various respondents was conducted based on the codebook. During the initial stages of the coding, items were coded unto the nodes as free nodes. However, as the coding proceeded, the relationship between nodes began to emerge, therefore these relationships were transformed into tree nodes. This continued until all the transcripts were coded. Afterwards, the nodes browser was reviewed to show the relationship between the various nodes. Queries were also run to obtain relations and main themes that emerged from the data in the form of axial relationship. Based on this relation, the data were reviewed and items coded selectively unto these nodes with the aim of developing a theory grounded on the dataset. At this stage hierarchical charts were drawn to explore the stakeholders’ perspectives about the feasibility and acceptability of the use of psychologists and health workers to provide school-based ASRH. The results are complemented with quotes from the transcripts.

## Results

### Provision of sexual and reproductive health information and Services in Schools

There was unanimity among all respondents about the need for school-based provision of SRH information and services. This approach was viewed as the most appropriate way of addressing challenges that adolescent face in trying to access SRH information and services. Participants noted that there was an increase in enrollment in schools and that many adolescents were in school. Further, they noted that students spend majority of their weekly hours in school. They therefore noted that school-based channels would be the most appropriate platform to disseminate SRH information. The following quotes buttress these points by respondents:

*“The most important place is school and at home. Because for especially these Christians you go to churches on Saturdays or Sundays. Isn’t it? Once a week. But school, at least 5 days in a week, so it the best place for educating adolescents on sexual and reproductive health issues”* (38-year married male parent, FGD).

*“…first of all, the statistics show that most of our young people are in school, so if you want to meet adolescents with information then you might as well go to where they can be found where most of them are. And if most of them are found in schools as the statistics are telling us, then it means that comprehensive sexuality education should start with the schools”*(Health Manager 1, IDI).

*“I also think that reproductive health services must be provided in schools. Children are exposed to a lot of information than we think. Many of them use the internet to seek information on certain things and the information they get may not be correct, so I think when the schools provide reproductive services, they will be informed on behavioral changes, positive behavioral changes that can help them to grow up as adults in future”* (Education Manager-2, IDI).

**Fig. 1:**
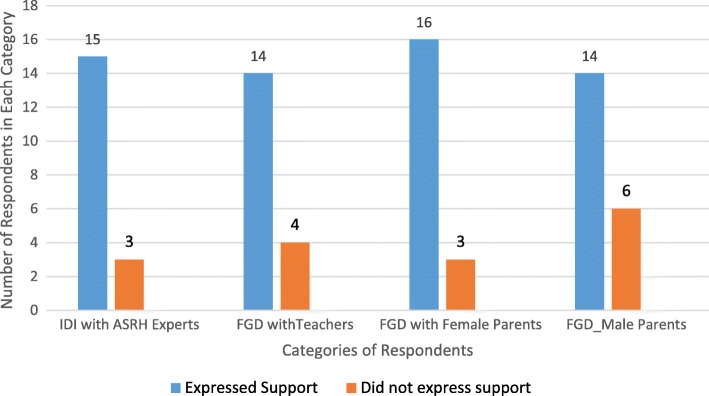
Respondents’ views on school-based sexual and reproductive health information and services

Parents, education managers and health workers all agreed that there was a need to extend the SRH services in schools and there were little variations in views across the various respondents. Out of the 18 key informants interviewed, 15 supported school-based ASRH services. In FGDs among teachers, 14 of the 18 participants also supported this strategy of providing ASRH service. In FGDs with parents, 16 of 19 female parents and 14 of 20 male parent support this approach. Figure 1 shows a graph depicting the respondents’ views on school-based ASRH services.

Some parents drew on their own experiences to highlight the negative effects of the lack of SRH information and services. One parent, for example, shared her experience with an unplanned pregnancy that led to her dropping out of school:

*“For me with my child when I realized his behavior, - he likes being in obscure places with young girls, I cautioned him not to impregnate someone. He should bear in mind that I gave birth at 15 years and for that matter I couldn’t continue with school, so the same thing might happen to any lady so he should be very careful”* (42-year female parent, FGD).

### Views on range of services to be provided in schools

**Fig. 2:**
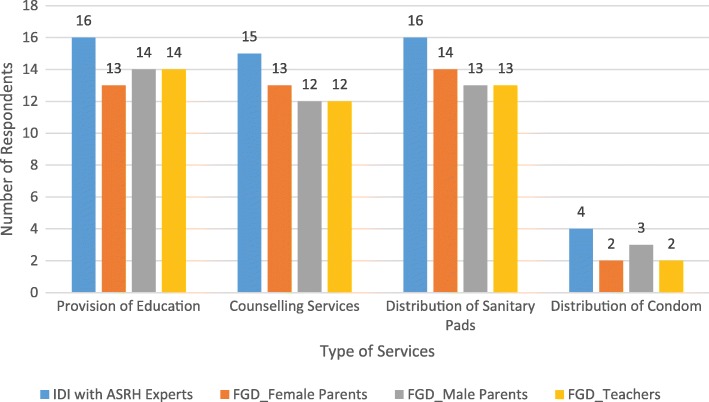
Respondents view on types of service to include in school-based ASRH

Participants mentioned health education, counseling services, and provision of sanitary pads as essential services for inclusion in any school-based adolescent health program. As shown in Fig. 2, there were mixed views on distributing condoms to students. Some respondents felt that it was appropriate to distribute condoms to students to enable them have protected sex should they wish to have sex. Those who held this view noted that some adolescents often engaged in unprotected sex and believed that the provision of condoms would prevent unplanned pregnancy and sexually transmitted infections. However, some respondents believed that distributing condoms would promote sexual activity as adolescents “experiment” with the condom. One respondent also noted that the current educational policy did not permit the distribution of condoms in school and suggested that condom distribution should be community-based. The following quotes from respondent illustrate these points:

*“….But in our schools we provide the counselling services, we can have the health corner alright where counselling services are provided, where materials are provided for reading, where we could have the sanitary towels or pads and other things. But for now I won’t subscribe to having condoms in school because of our socio-cultural background”* (Education Manger 4, IDI).

*“To me if they provide these services such as health education, distributing sanitary pad and condoms in school it is a good idea because nowadays if you advise the young ones not to have sex, they will have it. So it should be provided in schools for them to protect themselves”* (Male Parent, FGD).

*“My brother I told you from the very onset that for now Ghana Education Service doesn’t permit the provision of condoms in our schools. Do you get the point? We have a few though of health corners in our schools… But because our schools we don’t allow the provision of condoms, they don’t give condoms to the children in the school but however just as I said and I reiterate, if you have any condoms to provide let it be community-based”* (Education Manger 3, IDI).

Some respondents believed that it would be possible to revise the current prohibition of condom distribution within any school premise. Some education managers believed that if various stakeholders including parents were properly engaged it would be possible to obtain approval.

*“Although the current system does not allow the provision of some reproductive health service within school compound, if good consultation among parents, teachers, education managers is done, it will be possible to revise the policy*” (Education Manager 1, IDI).

### Perceived benefits of providing school-based sexual and reproductive health services

Most participants believed that school-based provision of SRH information and services would help adolescents make good SRH choices and reduce the prevalence of unplanned pregnancies, abortions, and sexually transmitted infections. Participants also noted that access to SRH information and services could reduce school dropout rate among females because those who become pregnant whilst in school are often compelled to drop out of school. The following quotes illustrate these points:

*“With the services, it will go a long way in reducing the rate of abortion and teenage pregnancy”* (Education Manager-1, IDI).

*“My view is they should provide sexual health services in schools, it is a world known issue now that we are having girls getting pregnant in schools but if these services were provided to them, I don’t think they will find themselves in this situations”* (Education Manager 2, IDI).

*“… it will help them delay becoming pregnancy, get pregnant when they want to, reduce the incidence of STIs and of course all these things will help them to improve their ability to stay in school and also improve their economic status in future”* (Adolescent Reproductive Health Expert, IDI).

In FGDs with students, they acknowledged challenges in accessing reproductive health services at the community level. In their view, students who seek such services at the community are often perceived as bad boys/girls and service providers are unfriendly to them. They therefore believe the delivery of these services at the school can increase access and break social barriers inhibiting the uptake of such services at the community as illustrated:

*“We cannot go to health facility to do family planning or go and buy condoms because the drugstore seller will say you are a bad girl or boy and may tell your parents. So, those who cannot abstain from sex do it without any protection and some become pregnant in the process. One of our colleagues had to stop school because she became pregnant. So having access to this in the school will help address this”* (17 years female student, FGD).

### Potential challenges in introducing comprehensive school-based ASRH

Feedback from participants suggested that there might be some challenges in introducing school-based SRH programs. Respondents identified resistance from religious organizations, faith-based schools, teachers and parents as a key challenge. This notwithstanding, some respondents believed that with extensive consultation it would be possible to have wider acceptance. The following quotes illustrate these differing views:

*“You see some churches will rise up against it. You know Catholics they are against the use of contraceptives, let alone providing the services at their school”* (Education Manager 1, IDI).

*“Yes, first is resistance from schools and from teachers but we need to do a lot of advocacy and some orientation for them to look at the benefits of such information before we rush into the school”* (Education Manager 3, IDI).

*“The only problem we’ll have is our conservative nature as Ghanaians. That’s the only challenge because, for instance, how will the parents feel if the child comes home to tell the mother that they shared condoms for us in school? Or they are doing family planning in schools. Every parent will be alarmed”* (Health Manager 3, IDI).

### Acceptability of the use of psychologists to provide ASRH services

**Fig. 3:**
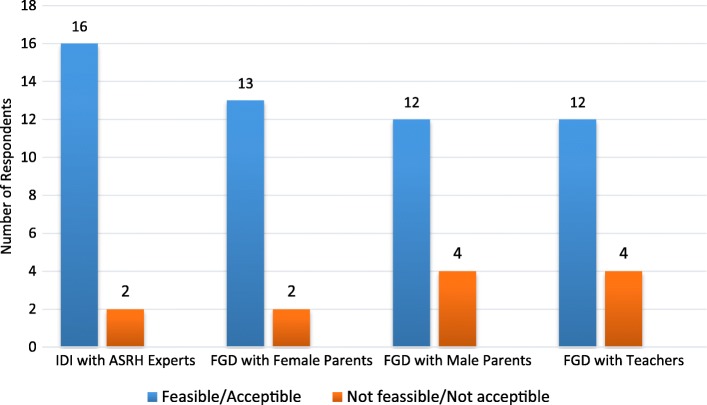
Respondents view on feasibility and acceptability of using trained psychologist to provide school-based enhanced ASRH

While there were dissenting views on the use of psychologists to provide school-based SRH information and services (Fig. 3), majority of the participants believed that it was feasible and acceptable to use them for these services to adolescents.

Those who believed that it was appropriate to have trained psychologists noted that using psychologists would enable teachers to focus on their primary responsibility of teaching. To them, even though the current education system allows for the training of teachers to serve as guidance and counseling officers at school, these teachers are not effective because they are still expected to teach. Some participants also observed that some students may not feel comfortable discussing sexual and reproductive issues with their teachers and noted that a neutral person, such as a psychologist, would be more appropriate. Other participants felt that psychologists were better placed to identify students experiencing emotional challenges and to offer timely support and counseling. The following quotes illustrates these views from respondents:

*“In our adolescent health program, we have a lot of psychology in it so they may not only provide the services and appropriate information but they are also able to provide the counselling that they need for children because they are many ramifications. A child may have some problem at home and that may manifest in inappropriate sexual behavior and we need such people to be able to delve into the details and be able to provide appropriate counselling for such kids”* (Education Manager 3, IDI).

*“I think it will be a great advantage for them (psychologists) to come in because they will add to whatever teachers currently provide. Because these kids we have they are very complicated and sometimes when teachers are saying what they know, they will say oh you are supposed to come and teach me science, you are supposed to come and teach me maths, how do you think that you know about sex or you know about the changes that occur to me”* (Male Teacher 2, IDI).

*“Our parents don’t have time or maybe they don’t feel comfortable teaching us certain things and in the schools too, the teachers may not be able to teach us everything but if we have the psychologists or when we have these experts in the school based on the education and the counselling it can help reduce this teenage pregnancies”* (Health Manager 2, IDI).

*“Several, quite apart from providing the information, if it so happens that some children have some challenges, you know, there will be the opportunity to intervene with any of the psychotherapy… yes the psychologist can intervene with some form of help, and give some therapy when necessary”* (Clinical Psychologist-1, IDI).

On the other hand, some respondents believed that using psychologists was not a feasible approach to addressing SRH challenges among adolescents largely because of the sustainability of such an approach. Specifically, respondents who were opposed to the use of psychologists generally raised concerns about the availability of trained psychologists and how to remunerate them for their services as illustrated:

*“And also knowing the number of schools in Ghana and the number of psychologists that will be in the system, I think visiting a school or sometimes they might not be able to visit the school in that case it would not be effective”* (Female Teacher, FGD).

*“The idea [use of psychologist] is good but the problem will be placement and who to employ them and pay for their services. You know psychologist come with some level of higher education and the money to pay them will be the problem”* (Male Teacher, FGD).

*“Training psychologists to visit schools is not sustainable…. So just use available structures, we have guidance and counseling coordinators there and what GHS [Ghana Health Service] does is that we train the guidance and counseling coordinators in adolescent sexual and reproductive health so most at times they sometimes even do the referrals to the health facilities”* (Health Manager 1, IDI).

*“Ehhh, we have to strengthen our counselling department, we have, GES [Ghana Education Service] has counselling department and those department should be strengthened, more psychologist and counsellors would have to be trained, well equipped with an in-depth knowledge in adolescent reproductive health so that we could refer students to that department for a redress*” (Headmaster, IDI).

### Use of health workers to provide school-based SRH information and services

**Fig. 4:**
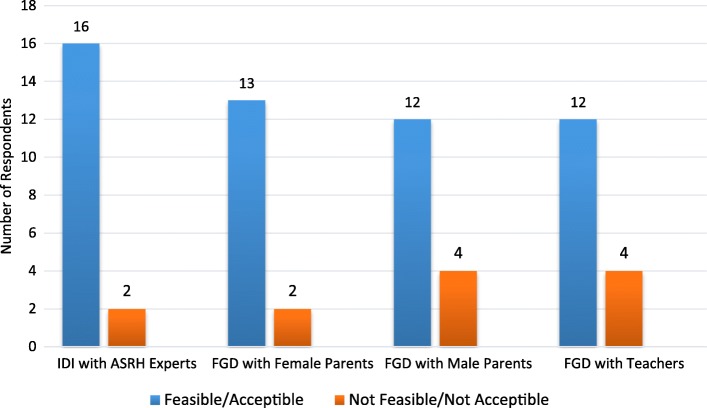
Respondents view on feasibility and acceptability of using health workers to provide school-based enhanced ASRH

The results of the study also showed that stakeholders had a positive view on the use of health workers to deliver SRH services in school for adolescents (Fig. 4). According to respondents, using health workers would ensure that adolescents have access to accurate SRH information as well access to services. Using health workers was also perceived as a strategy that could be helpful in linking school-health services to the mainstream health care system for easy referral. These views are illustrated in the following quotes:

*“Yes, I agree that health workers should visit schools to talk to the children about sexual and reproductive health. It will help them very well because the health workers can easily refer a student with a problem to hospital to get quick care”* (Female Parent, FGD).

*“You know that is the work of health workers. They have been trained and they have knowledge on sexual and reproductive health. So it would good to use them”* (Male Parent, FGD).

*“When we have this adolescent health centers with experts going there to work is very easy, for instance we have Nima, see Nima Cluster of schools for instance if we can establish one there, another cluster of school, Dansoman. You see, when we have these adolescent health centers it will rather help the nation, it will help Ghana, it will help Accra Metro”* (Education Manager 1, IDI).

### Views on operationalizing the use of psychologists and health providers to deliver school-based SRH services

Study findings underscored the need to address gender-related issues when operationalizing any school-based SRH programs. In FGDs with teachers, parents and students, it emerged that it would be preferable for male service providers to be assigned to male students and vice versa for females. According to respondents, matching the sex of service providers to the target students would increase students comfort in discussing sensitive SRH issues. Religious and cultural reasons for sex-separated programs were also highlighted. These views are illustrated in the following quotes:

*“For our Islamic religion, what we are supposed to do is we have the females, we don’t mix the females and the males. So we have to have a psychologist for the female and the psychologist for the male”* (46-years Teacher, FGD).

*“Madam like, they should separate the boys from the girls so that people can ask questions. When they are mixed, people feel shy to ask some questions which ermm is bothering them. So when they separate the boys from the girls’ people will be able to ask questions and will be given a solution to all their problems”* (15-years female student, FGD).

Female students were more concerned about separating male and female students. Female students reported that male students often make fun of them when female reproductive and sexual organs are mentioned. In addition, female students believed that female teachers and service providers have experienced what they may be going through and would therefore be in a better position to explain and provide support to them. The following quotes illustrates these points:

*“A female teacher, because she is a woman and she has been what you are going through now. And she will more have knowledge of teaching”* (17 years female student, FGD).

*“…the boys they like when the teacher is teaching and will be saying, they will be saying vagina and those things about females. The boys will be happy and they will be laughing”* (16 years female student, FGD).

The results of the study also showed that to be able to implement such a system will involve some work with the existing bureaucracies and good collaboration with various stakeholders including the Ministry of Education, Ghana Health Service and development partners who are currently engaged in SRH services and education in order not to run into challenges.

*“As I said if you do not use the right channels the heads may not allow because they may think you are interrupting their contact hours and those would be the challenges. They will not be ready to give you access to the people so if you use the right channels I don’t think you will have any problem*” (Headmistress, IDI).

*“This is [a] policy issue and will require bringing all stakeholders together to discuss. If Ghana Education Service make it a policy, than nobody can say no to it. We will implement it”* (Teacher, IDI).

The study further found that one approach that could be used to implement school-based adolescent SRH is to organize schools into clusters and have one psychologist or health worker assigned to a specific cluster. This design was suggested to address the challenge of finding an adequate number of trained psychologists. The psychologist could provide age-specific education to a group of students and one-on-one counselling for those with specific psychological problems. These suggestion is illustrated in the following quote:

*“What can be done is to group schools, a number of schools or students that can meet at a center, then a trained psychologist can meet with about two hundred, three hundred, five hundred students in a big hall giving the same information at the same time so by that the target groups can be met, I mean the number of children that he can meet will expand and will be more”* (Education Manager 2, IDI).

*“Psychologist, how many are in the system? We may have to put schools together where you assign on psychologist in-charge”* (Education Manager 4, IDI).

*“The psychologist can educate a group of students on psychological changes and sexual and reproductive health and can provide counselling to individual students or do group counselling for people with similar problems”* (Clinical Psychologist 2, IDI).

Further, stakeholders noted that the use of trained psychologists and health workers for the provision of adolescent and sexual health would require making some changes to the school timetables to cater for the time that would be used to provide such services. Further, some respondents highlighted the need to identify an appropriate service point or room where such services would be provided as privacy is required to assure adolescents that information provided would be treated as confidential. To implement this system of school-based provision of SRH would also require changes in existing policy and acquiring logistics that would be used in the delivery of the service.

*“It will not be easy because sometimes the school will tell you they already have the time table for the term. So maybe giving you that enough time they [school] will not agree”* (Health Manager 1, IDI).

*“….You would have to start from the policy level come to the management and then it transcends to the school level. So the challenge is that you must have the materials that you would use for the education ready, err if you have any teaching and learning materials all these should be ready and it should be down to earth to reach the target group”* (Education Manager 6, IDI).

*“There should be a space or a room for them. So maybe there wouldn’t be availability of a place for them to work. And then maybe we need to train more of the health personnel because this require extra training to deal with this thing. There should be [an] adolescent nurse, a nurse who… a guru, somebody who knows adolescent health, who has been trained, had extra training on adolescent health to tackle them. So maybe shortage of staff will not allow us to err…reach our goal”* (Health Manager 3, IDI).

### The paradigm of school-based SRH programs using psychologists and health workers

**Fig. 5:**
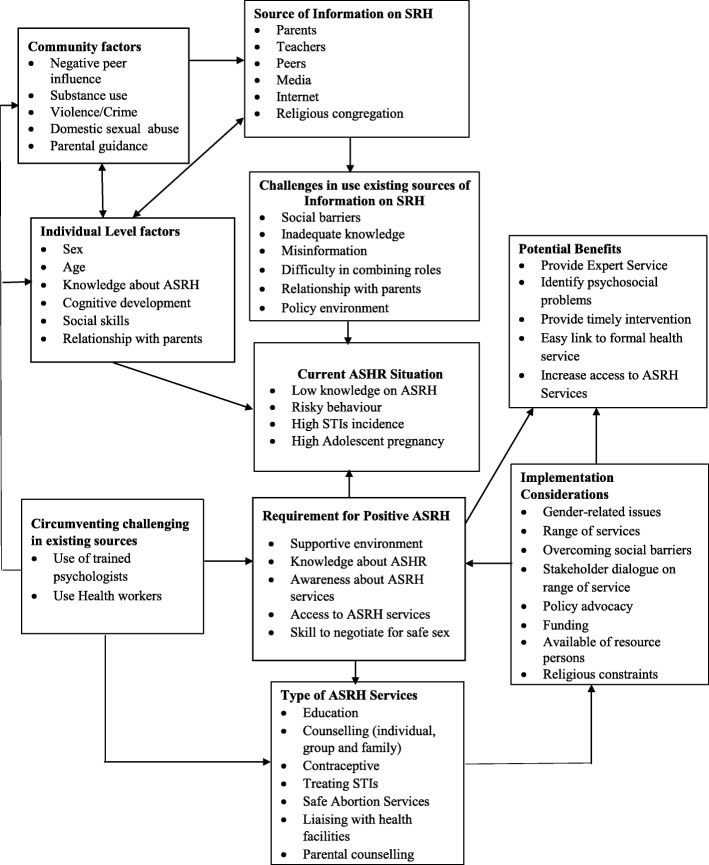
Framework on use of trained psychologists and health workers in providing school-based ASRH Services

Figure 5 is a framework developed from the data to explain the contextual issues in using health workers and psychologists to provide SRH services in schools. The study showed that existing sources of SRH information such as parents, teachers, peers, electronic media, community members, churches and mosques had practical challenges. Social norms especially made it difficult for parents and teachers to freely provide these services. The existing guidance and counseling services in school, which serve as channels for providing SRH education and support were being undermined by competing roles as the counselors still had to perform their primary role as teachers. The effect of the lack of access to SRH services is low knowledge among adolescents, high adolescent pregnancy and STIs. Adolescent generally need a positive SRH environment, high knowledge on sexuality, and access to SRH services to able to make informed decision which is not the case in Ghana. To that end, respondents generally believed existing systems were not achieving their objective and felt that it would be appropriate for both trained psychologists and health workers to provide school-based SRH to adolescents. In that opinion, using psychologists and health workers can create this positive environment and increase access to ASHR services. The use of health workers and trained psychologists was viewed to have additional benefits. Specifically, psychologists would be better able to identify and support adolescents with psychosocial problems, while health workers foster referrals to SRH services by creating a link between the school health service and formal health system delivery as diagrammatically presented in the figure. The types of services will include providing health education to various classes, individual counselling for those with psychological and physical problems. Group counselling sessions and family counselling could also be organized for students only and student who have family problems respectively (Fig. 5).

However, stakeholders perceived a number of practical challenges around how this could be implemented. Key among the challenges were how to pay for the services that health workers and trained psychologists would render, and availability of psychologists to cater for all schools. Another important challenge raised was around the acceptability of certain services such as distributing condoms and provision of contraceptives services especially in faith-based schools. Nonetheless, respondents believed that garnering enough support from parents and other stakeholders could help overcome this challenge. In designing school-based SRH programs that would rely on trained psychologists and health workers, our finding underscored the need for gender-sensitive programs that would enable both boys and girls to feel comfortable discussing sensitive issues. Once this is implemented, it is go to affect individual and community level factors that predispose adolescents to negative behavioral tendencies with its associated consequences.

## Discussion

### Acceptability of school-based SRH services

The study showed that stakeholders perceived the provision of SRH services in schools as laudable and an idea worth implementing. There was general consensus among all stakeholders, including parents, about the need to extend SRH services to school. Previous studies have demonstrated the importance of comprehensive sexuality education. An earlier study in the United States found that adolescents who received comprehensive sex education in school were significantly less likely to report teen pregnancy than those who did not receive formal sex education [47]. In Indian, the introduction of sexual and reproductive intervention in school was also found to have resulted in a significant increase in the knowledge in both boys and girls. In that study, among girls, the percentage of poor knowledge reduced significantly from 64.1% (pre-intervention) to 8.3% (post-intervention) and among boys from 37.7% (pre-intervention) to 3.5% (post-intervention). Similarly, increase in knowledge level was also observed in various other aspects of reproductive and sexual health including, STI, HIV/AIDS and perceptions about premarital sex [48].

Although majority of respondents supported adolescents’ access to comprehensive SRH information and services, they generally disapproved of providing contraceptive services in school because of concerns that contraceptive provision would promote sexual activity. Nonetheless comprehensive programs have been reported to be more beneficial. In a review of 56 adolescent SRH programs, it was found that abstinence programs did not delay initiation of sex but two third of comprehensive programs had positive impact on sexual behavior including delay in initiation, use of contraceptives and condoms [49]. Drawing from the findings of this study, it would be important for any discussions on designing a plan for school-based services to go beyond promoting abstinence only to making it possible for adolescent to access services that can ensure that adolescents who wish to engage in sex do that with protection against pregnancy and STIs.

Though distributing condoms and providing contraceptive services to adolescents was unacceptable to majority of respondents in this study, results suggested that it would be possible to garner more support for contraceptive service provision through extensive stakeholder engagement. Contraceptive service provision in schools may also require further studies to explore view of health workers on their attitude towards providing contraceptives services to adolescents as a study in Nigeria found that many healthcare providers have unfavorable attitudes towards the provision of contraceptives for unmarried adolescents [50].

### Changes required to implement school-based SRH services

Though stakeholders in this study believed it was acceptable and feasibility to introduce school-based SRH programs delivered by psychologists and health workers, the implementation of such programs would require further consultation to inform the operationalization of the programs. Studies suggest that programs to promote access to and uptake of adolescent SRH services are reported to be most effective when adolescent-friendly facility-based approaches are combined with community acceptance and demand-generation activities [29]. In South Africa for example, a pilot study on school-based SRH services was found to be acceptable in the community and feasible with scale-up [51]. Ghana can draw on this experience to pilot this system in selected institutions where lessons learnt can then inform scale up. Locating health services in schools or providing outreach services has the potential to reduce transport costs, increase accessibility and provide links between schools and communities [52]. Moving forward, there is the need to organise stakeholders to deliberate on the findings of this study and fashion out an implementation plan. During the pilot phase there will be the need for an implementation research to inform scale up [53, 54].

Our findings also underscore the importance of allocating time for school-based ASRH services as there is need for enough time for interaction between the service provider and the students. In addition, schools must ensure that they identify a private space where the services can be provided. Importantly, school-based SRH service provisions requires a change in policy as the existing policy on school health does permit the inclusion of contraceptive services.

### Views on the use of psychologists and health workers for delivery SRH services

The study showed that majority of the respondents perceived a system that will make it possible for sexual and reproductive services to be delivered in schools by trained psychologists as essential as the present system was not meeting the needs of adolescents. Teachers who are in-charge of guidance and counseling services were reported to perform those roles in addition to their primary role (teaching) and were less effective in guidance and counseling. Further, students were described as being unwilling to discuss sensitive issues with teachers. The study also showed that some adolescents might not be comfortable discussing sexual issues with their teachers, as they may be perceived to be immoral. When service providers are judgmental towards adolescents who seek services they may feel reluctant to go back due to shyness [55]. As such, ‘neutral’ service providers such as psychologists and health workers may increase young people’s use of SRH services. Psychologists were also viewed to be better placed to detect emotional distress and offer timely counseling. For the purpose of social and cultural correctness, teachers were viewed as not been the right people to provide such information. The perception was that some teachers are likely to even take advantage of the adolescents and engage in sexual acts with them. Therefore, parents perceived finding a neutral person who will only visit to provide such information and services as a better option. In a systematic review of youth friendly programs, it was found that accessibility of health care; communication; staff attitude; medical competency; guideline-driven care; age appropriate environments; youth involvement in health care; and health outcomes were seen as core to the youth [56].

We found that stakeholders believed that using psychologists would also be beneficial in providing support to adolescents with diverse psychosocial needs. It is well documented that adolescents who exhibit delinquent behaviors are more likely to engage in risky sexual activities [56–59]. These behaviors often have psychological undertones that may be more accurately identified by psychologists who can then provide appropriate interventions.

Further, using health workers was believed to enhance linkages with the health system. A study in the United States showed that using a school nurse to deliver intervention aimed at encouraging adolescents to stop smoking was effective as intervention condition participants were almost twice as likely to be abstinent per self-report at three months compared with control participants [60]. In another study it was found that using school nurse to provide contraceptive service increased contraceptive use [61]. A study in Ethiopian also found that majority of the health workers had generally positive attitudes toward SRH to adolescents and this was reported to enhance the provision of services to adolescents [62]. Building from the evidence above, it is important to reflect carefully on using psychologists and health workers to deliver ASRH services in schools as it could contribute immensely to improving young people sexual and reproductive lifestyle. Opportunities therefore exist in the use of psychologists though now study has documented the use psychologist in providing adolescents SRH. The use of psychologists will be a novel intervention as using trained health workers have been widely reported in the studies referred to earlier.

### Limitation of study

Although this study provides evidence on stakeholders’ views about using health workers and psychologists to provide school-based adolescent SRH services, it is important to situate the conclusions in the context of one limitation. This study was conducted in one region in Ghana on a sample that is not representative, hence the findings cannot be generalized. This notwithstanding, in conducting the study, we followed the methodological requirements for a qualitative research such as the RATS checklist [63], Consolidated criteria for reporting qualitative research (COREQ), [64] and acceptable practice in fieldwork, analysis and interpretation [65].

## Conclusions

Our study findings show that there is widespread acceptance of the need for enhanced school-based SRH programs as the present systems are not perceived to sufficiently meet adolescents’ SRH needs. Results demonstrate that it is acceptable to use trained psychologists and health workers to deliver school-based SRH information and services in the Ghanaian school context. However, provisions must be made to cater for financial and other logistical considerations in the implementation of school-based SRH programs that rely on external human resources.

## Abbreviations

AMA: Accra metropolitan assembly
ASRH: Adolescents sexual and reproductive health
CSE: Comprehensive sexual education
FGD: Focus group discussion
ICPD: International conference on population and development
IDI: In-depth interview
PTA: Parent teachers association
SRH: Sexual and reproductive health
STIs: Sexually transmitted infections
